# Why do I use generative artificial intelligence (GenAI) to seek health information? A perceptual perspective of GenAI users

**DOI:** 10.3389/fpubh.2026.1827765

**Published:** 2026-05-22

**Authors:** Hui Zhu, Jianfei Ding

**Affiliations:** School of Marxism, Anhui Medical University, Hefei, China

**Keywords:** GenAI users, generative artificial intelligence, health information seeking intention, internet plus healthcare, perceived characteristics

## Abstract

**Introduction:**

China’s vigorous advancement of the “*Internet Plus Healthcare Services*” initiative, as a core component of social security system optimization for improving residents’ health welfare, furnishes a robust impetus for the integration of Generative Artificial Intelligence (GenAI) within the healthcare sector, particularly in the domain of health information provision, where GenAI is widely acknowledged to harbor substantial transformative potential. Nevertheless, empirical research specifically probing the modalities through which users adopt GenAI for health information seeking remains limited in extant scholarly literature.

**Methods:**

This study garnered primary data via a structured online survey and employed partial least squares structural equation modeling (PLS-SEM) to dissect the antecedent factors and underlying mechanisms governing users’ health information seeking intention via GenAI, grounded in the perspective of user perceptions.

**Results:**

PLS-SEM results indicate that user perceptions of GenAI (perceived competence, perceived convenience, and perceived anthropomorphism) positively influence on both user trust in GenAI and subjective norms, which in turn positively affect users ‘health information seeking intention through GenAI. Moreover, digital health literacy significantly moderates the relationship between user perceptions of GenAI and their health information seeking intention.

**Discussion:**

These findings yield valuable empirical insights for facilitating the optimized and scaled adoption of GenAI in health information services, enhancing the public health output of digital health policies, improving residents’ health welfare, and further alleviating the operational burdens borne by traditional healthcare resources.

## 1 Introduction

In recent years, China has successively introduced a series of policies, including *Healthy China 2030 Plan*^1^ and the Internet Plus Healthcare initiative,^2^ and identified digital health services as a key measure to improve the social security system and enhance residents’ health welfare. Health information refers to all types of information related to physical and mental health, disease prevention, nutrition, and wellness maintenance. Traditionally, the public has primarily sought health information from medical professionals (1). However, the rapid advancement and widespread adoption of digital technologies have fostered a new paradigm of health information-seeking that integrates intelligent technologies intrinsically. Among them, generative artificial intelligence (GenAI) represents one of the most promising modes for acquiring health information (2, 3). GenAI refers to a category of artificial intelligence models capable of generating new content based on patterns learned from large-scale training data. Unlike traditional rule-based or discriminative AI systems, GenAI leverages advanced architectures such as large language models to produce human-like and contextually relevant content. In the medical and healthcare field, GenAI has rapidly evolved from a niche technological innovation into a widely accessible tool for providing health information. This trend is mainly driven by the maturity of publicly available models such as ChatGPT and DeepSeek, as well as the growing public demand for instant, low-cost health advice. Endowed with distinctive advantages—including providing real-time responses to intricate queries (4), generating personalized content tailored to individual needs (5), and offering cost-effective, round-the-clock health knowledge services (6), GenAI has transcended the inherent limitations of traditional health information-seeking approaches. By enabling the public to obtain reliable health information without direct medical consultation, GenAI can effectively divert non-urgent health inquiries away from grassroots healthcare facilities, thereby reducing their consultation pressure. This function directly alleviates the operational burden on traditional medical resources and aligns with the core objective of the Internet Plus Healthcare initiative to optimize the social security system.

Unlike conventional health information platforms, GenAI is capable of deeply deciphering users’ latent needs and delivering customized, structured, and readily comprehensible health advice. This salient feature substantially lowers the threshold for the public to access professional health information. Accurate and accessible health information can encourage the public to adopt evidence-based and appropriate health behaviors, thereby providing a robust impetus to China’s vigorously promoted Internet Plus Healthcare initiative and the national Healthy China strategy. The implementation of these policies and empowerment of technologies have not only promoted the digital transformation of health information services, but also directly improved health accessibility for residents—especially vulnerable groups. These efforts have exerted a profound impact on enhancing the public’s mental health, life satisfaction, and sense of happiness, providing a new practical path for China’s social security system to improve residents’ health welfare. The global AI-driven health information service market is currently undergoing a period of explosive growth. Industry projections indicate that the scale of the global artificial intelligence market will surpass $1.3 trillion by 2032, underscoring the immense economic value embedded in this domain (7). During the COVID-19 pandemic, the suspension of offline medical services and prevalent public concerns regarding virus transmission expedited the migration of health information-seeking channels from traditional offline venues to online platforms. This paradigm shift in consumption and information-seeking behaviors has endured into the post-pandemic era, further catalyzing the application and popularization of GenAI in the health information sector.

However, for GenAI users, the rapid market expansion is accompanied by a series of challenges, including concerns over accuracy, requisite digital skills, and access barriers (8). The internal operational mechanisms of GenAI remain opaque, and the accuracy of its outputs cannot be guaranteed (9). In the context of health information provision, the proliferation of inaccurate and high-risk content and the hallucination risks inherent to GenAI have negatively impacted users’ intention to adopt this technology (10–12). Public trust in GenAI—particularly regarding complex health-related issues—remains relatively low (13). In addition, potential risks such as privacy breaches, algorithmic bias, and inconsistent professionalism in intelligent responses have aroused widespread concern among users (14, 15). For individual users, these multifaceted factors collectively influence their intention to use GenAI for health information seeking, leading to significant heterogeneity in usage behavior. Empirical evidence indicates that medical experts and online search engines remain the most prevalent sources of health information, while the adoption of GenAI in this domain remains limited (16). Therefore, exploring the key antecedents and internal mechanisms that drive users to adopt GenAI for health information seeking is of great significance for promoting the sound development of the GenAI health service industry, meeting the public’s diversified health information needs, helping China’s social security system improve residents’ health welfare through digital health services, and deepening the public health outcomes of the *Internet Plus Health Services* program.

Existing research on users’ health information-seeking intention or behavior has predominantly explored its influencing factors from the lenses of users’ individual attributes, online platform features, and broader contextual factors. Users’ internal perceptual constructs (e.g., performance expectancy, perceived susceptibility, effort expectancy, perceived benefits, perceived severity, perceived barriers, and self-efficacy), health literacy, habits, and hedonic motivation all exert significant impacts on users’ adoption intention toward health information-seeking tools (16–19). On the platform dimension, relevant studies have focused primarily on the inherent attributes of platform information and the features of access channels—such as information practicality, perceived credibility, platform reliance willingness, and technical specifications—which jointly determine individuals’ propensity to select a specific channel for health information acquisition (20, 21). Contextual factors that modulate individuals’ health information-seeking intention encompass disease stigma, norms governing information-seeking behaviors, social influence, and cultural underpinnings (22, 23). Notably, a subset of studies has posited that health literacy and social influence exhibit no significant correlation with users’ behavioral intention to adopt generative AI for health information-seeking purposes (16).

However, extant studies have largely focused on users’ health information-seeking behaviors via conventional online tools, while overlooking the impacts of GenAI’s unique perceived attributes on users’ health information-seeking intention. Prior applications of the unified theory of acceptance and use of technology (UTAUT) and technology acceptance model (TAM) in health information contexts have primarily examined generic constructs such as perceived usefulness and ease of use (16, 17). Yet they have largely neglected GenAI-specific attributes such as anthropomorphism and instantaneous responsiveness that differentiate GenAI from conventional search engines and health apps (6). With its distinctive advantages in interactivity, intelligence, personalization, and convenience, GenAI renders users’ perceived experience a core antecedent of their usage intention. In the context of GenAI-powered health information services, users’ active perceptions of the technology’s core attributes directly shape their trust in the disseminated information and subsequent usage behaviors. Therefore, it is imperative to integrate the user perception perspective into the research framework of GenAI-mediated health information-seeking behavior, address the gaps in the existing literature, and deepen the understanding of the mechanisms underpinning users’ adoption of intelligent technologies for health information seeking.

To address this research gap, the present study focuses on the impacts of users’ perceptions of GenAI’s core technical attributes on their health information-seeking intention. Specifically, this study examines the effects of three key perceived attributes—perceived competence (PCP), perceived convenience (PCV), and perceived anthropomorphism (PAN)—on users’ willingness to seek health information via GenAI. Furthermore, we test the mediating roles of trust in GenAI and subjective norms (SN) in the aforementioned relationships. Meanwhile, this study explores the moderating effect of digital health literacy (DHL) on the link between users’ perceptions of GenAI and their GenAI-enabled health information-seeking intention (HISI).

This study makes three key contributions to the extant literature. First, it extends the research agenda on how GenAI’s perceived technical attributes influence users’ health information-seeking intention. Existing studies rarely systematically delineate the core perceived technical dimensions of GenAI, nor do they investigate the differential effects of these dimensions on users’ information-seeking intention in the highly sensitive and professional context of health services. By focusing on three core perceived technical attributes among Chinese users and their impacts on health information-seeking intention, this study enriches the scholarly discourse on user technology acceptance and health information behavior within the domain of digital health communication. Second, this study further unpacks the specific mechanisms through which GenAI’s perceived technical attributes affect users’ health information-seeking behaviors, aiming to address the limitation that existing studies have overlooked the potential mediating pathways between technology perception and health information-seeking intention. By systematically examining the mediating roles of trust in GenAI and subjective norms, this study refines the theoretical model elucidating the driving mechanisms of users’ behavioral intention in GenAI-enabled health information contexts. Finally, this study investigates the moderating effect of digital health literacy on the relationship between users’ perceptions of GenAI and their health information-seeking intention. This inquiry may provide a novel perspective for reconciling the inconsistent findings reported in the literature regarding the link between technology perception and users’ information-seeking behavioral intention.

## 2 Hypothesis development

### 2.1 User perception and health information seeking intention

Drawing on the Unified Theory of Acceptance and Use of Technology (UTAUT), the perceived usefulness and ease of use of emerging technologies exert a notable positive impact on the frequency of technology adoption, which in turn fosters the development of users’ behavioral intentions (24). The rationale for GenAI being regarded as a “potential ally” in the healthcare service domain stems from its acknowledged functional capabilities and the perceived accuracy of the health information it disseminates (25). Perceived competence is defined as users’ subjective perception of the professionalism and efficiency demonstrated by GenAI in executing health-related tasks (26). This construct encapsulates dimensions of intelligence, competence, technical proficiency, and operational efficiency (27). The stronger users’ perceived competence of GenAI, the more pronounced their intention to utilize this technology for health information-seeking purposes. Perceived convenience is recognized as a pivotal driver of users’ GenAI adoption, as well as a salient factor influencing their health information-seeking intention (17, 28). Perceived anthropomorphism reflects GenAI’s human-like interactional features, which can readily elicit users’ emotional resonance, mitigate their sense of unfamiliarity with intelligent systems, and enhance the overall user experience of interaction. Notably, familiarity and interactive enjoyment constitute core user-valued attributes that shape the adoption intention of digital health tools (29, 30). Extant empirical studies further indicate that the anthropomorphic traits of artificial intelligence can exert a positive influence on users’ adoption intention and facilitate the uptake of GenAI applications in practical scenarios (7, 31). Based on the above analysis, the following hypotheses are proposed:

*H1*: Perceived competence positively affects HISI.

*H2*: Perceived convenience positively affects HISI.

*H3*: Perceived anthropomorphism positively affects HISI.

### 2.2 The mediating role of trust

Trust is conceptualized as users’ attitudinal orientation toward GenAI. Trust in GenAI is manifested in positive expectations and evaluations of the technology’s performance, operational processes, and intended purposes, as well as the willingness to accept potential vulnerabilities and assume risks based on health recommendations provided by GenAI (5, 32). When users hold stronger perceptions of GenAI’s perceived competence, perceived convenience, and perceived anthropomorphism, they are more inclined to recognize the accuracy and professionalism of the health information generated by the technology, thereby fostering a sense of reliance and trust in GenAI. Extant studies have demonstrated that the anthropomorphic traits of artificial intelligence can exert a salient positive effect on user trust (33). In an empirical investigation of the antecedents of consumer trust in AI chatbots, Li et al. (34) further verified that anthropomorphism exerts a significant impact on user trust. Trust constitutes a critical antecedent of users’ willingness to adopt AI-enabled tools; specifically, users with higher levels of trust in GenAI are more likely to use it for health information seeking (35, 36). Therefore, we propose:

*H4a*: Perceived competence positively influences HISI through trust.

*H4b*: Perceived convenience positively influences HISI through trust.

*H4c*: Perceived anthropomorphism positively influences HISI through trust.

### 2.3 The mediating role of subjective norms

Subjective norms are defined as the extent to which individuals perceive that significant others believe they ought to adopt a specific system or technology (37). In the early adoption phase of intelligent tools, external validation tends to take precedence over personal experiential knowledge (38). When users hold favorable perceptions of GenAI’s competence, convenience, and anthropomorphism, they are more susceptible to the endorsement and recommendations of family members, friends, and other significant stakeholders. Consequently, such users perceive greater external normative pressure to utilize GenAI for health information-seeking purposes. In other words, users’ positive perceptions of GenAI exert a positive influence on their subjective norms regarding GenAI-based health information seeking. Furthermore, subjective norms have been empirically verified to exert a direct impact on users’ behavioral intention to adopt GenAI-powered chatbots (39). Based on the above, we propose:

*H5a*: Perceived competence positively influences HISI through subjective norms.

*H5b*: Perceived convenience positively influences HISI through subjective norms.

*H5c*: Perceived anthropomorphism positively influences HISI through subjective norms.

### 2.4 Digital health literacy as a moderator

Digital health literacy is conceptualized as an individual’s capacity to search for, access, comprehend, and critically appraise health information, as well as to apply the acquired knowledge to effectively address health-related issues. Within the digital context, DHL specifically denotes the proficiency to execute the aforementioned competencies in digital environments (16). The impact of GenAI’s perceived attributes on users’ intention to adopt the technology for health information seeking may be contingent on their level of digital health literacy. To date, no direct empirical evidence has been documented to confirm that DHL moderates the relationship between GenAI’s perceived characteristics and health information-seeking intention. Nevertheless, extant research indicates that individuals with higher DHL levels exhibit a stronger propensity to adopt generative artificial intelligence applications (23). Although the general public is increasingly reliant on GenAI for health-related services, users with low DHL cannot often screen health information critically and to comprehend or operationalize the technology effectively. Even if such users recognize the technical merits of GenAI, they may struggle to accurately evaluate the technology’s practical value, thereby attenuating the positive effect of GenAI’s perceived attributes on their usage intention. In contrast, a high level of DHL enables users to conduct a comprehensive and rational assessment of GenAI’s core attributes, which in turn amplifies their intention to use GenAI for health information-seeking purposes. In other words, among users with high digital health literacy, positive perceptions of GenAI’s technical characteristics are more likely to be translated into actual health information-seeking intention. Therefore, the following hypotheses are proposed:

*H6a*: Digital health literacy strengthens the relationship between PCP and HISI.

*H6b*: Digital health literacy strengthens the relationship between PCV and HISI.

*H6c*: Digital health literacy strengthens the relationship between PAN and HISI.

## 3 Data and methodology

### 3.1 Sample and data collection

This online survey was conducted in December 2025, targeting individuals with a basic understanding of generative artificial intelligence (GenAI). Respondents were required to understand how to obtain health information through generative artificial intelligence and possess basic relevant knowledge. Participants who did not meet the criteria were excluded from the survey. Before the formal survey, the research team disseminated the survey link via the WeChat platform and recruited over 50 participants to complete a pilot study. Participants were instructed to fill out the draft questionnaire, assess each measurement item in terms of semantic coherence, logical consistency, and potential ambiguity, and provide constructive feedback for item revision. Based on the collected feedback, several measurement items were revised and refined to ensure the questionnaire’s logical rigor and readability. For the formal survey, Wenjuanxing,^3^ a professional online questionnaire platform, was adopted as the official distribution channel. This study adopts convenience sampling based on an online participant pool, which may lead to coverage bias. The sampling frame cannot fully represent the overall population of GenAI users in China, and potential undercoverage of certain subgroups is inevitable. To avoid duplicate submissions, the platform was set to permit only one response per participant via the exclusive survey link. In addition, all questionnaire items were designated as mandatory to eliminate missing values in the collected data. After rigorously excluding invalid questionnaires (e.g., incomplete responses, random answering), a total of 420 valid questionnaires were obtained, which served as the final sample for subsequent empirical analysis.

The demographic characteristics of the sample are presented in Table 1. The gender distribution was nearly balanced, with males accounting for 49.8% and females 50.2% of the participants. Some 34.3% of respondents were married, and nearly 58% fell within the 18–25 age group. Moreover, 22.9% possessed a bachelor’s degree or above, and 85.48% of participants self-rated their physical health as healthy or very healthy.

**Table 1:** Demographic profile of respondents.

Characteristic	Demographic	Frequency	Percentage (%)
Gender	Female	209	49.8
	Male	211	50.2
Age (years)	18 and below	10	2.4
	18–25	243	57.9
	26–45	137	32.6
	46 and above	30	7.1
Educational level	Junior middle and below	17	4.0
	Senior high	15	3.6
	Junior college	292	69.5
	Bachelor degree and above	96	22.9
Health status	Vigorous	96	22.86
	Healthy	263	62.62
	Average	57	13.57
	Poor	4	0.95
Marital status	Single	276	65.7
	Married	144	34.3
Family income (monthly)	￥5,000 and below	60	14.3
	￥5,000–￥10,000	116	27.6
	￥10,000–￥15,000	91	21.7
	￥15,000–￥20,000	76	18.1
	￥20,000 and above	77	18.3
Frequency of use	Frequent	32	7.6
	Often	97	23.1
	Occasionally	230	54.8
	Hardly ever	42	10.0
	Never	19	4.5

The number of samples is 420.

Source(s): Authors’ own work.

### 3.2 Measurements

The questionnaire comprised two sections: the first collected respondents’ basic demographic characteristics, while the second measured the core constructs of the study, namely perceived competence, perceived convenience, perceived anthropomorphism, trust in GenAI, subjective norms, digital health literacy, and GenAI-enabled health information-seeking intention.

All measurement items were adapted from established mature scales and appropriately modified to fit the research context. To ensure linguistic and cultural equivalence, a systematic translation and adaptation procedure was conducted. First, two bilingual researchers independently translated the original English scales into Chinese. Second, a third researcher synthesized the two translations into a preliminary Chinese version, which was then back-translated into English by two native English-speaking translators who were blind to the original items. The back-translated versions were compared with the original scales by the research team, and discrepancies were discussed and resolved iteratively until semantic equivalence was achieved. During this process, we conducted appropriate cultural adaptation. For instance, we adjusted the expressions to align with Chinese users’ habits of interacting with artificial intelligence and the practical usage context in China. Third, an expert panel comprising three scholars in health communication and one practicing physician reviewed the Chinese version for content validity and cultural appropriateness. Specifically, the perceived competence scale was sourced from Zhou et al. (40), perceived convenience from Qiu et al. (41) and Liang and Shi (42), perceived anthropomorphism from Priya and Sharma (43) and Al-Emran et al. (44), trust from Choudhury and Shamszare (5), subjective norms from Lai and Li (45), digital health literacy from Liu et al. (46) and Zhao et al. (47), and GenAI-enabled health information-seeking intention from Ling et al. (48). A 5-point Likert scale, anchored at 1 = strongly disagree and 5 = strongly agree, was employed to rate all items measuring the aforementioned constructs.

### 3.3 Technical analysis

Partial least squares structural equation modeling (PLS-SEM) was adopted as the primary analytical technique in this study, with its selection justified by three key considerations: First, PLS-SEM is particularly adept at analyzing complex models incorporating multiple constructs, mediating variables, and moderating variables—a critical advantage given the eight constructs included in the present study. Second, it does not impose stringent requirements for data normality, which enhances its flexibility for empirical research. Third, PLS-SEM boasts high statistical power and applies to both exploratory and confirmatory research, thereby yielding robust explanatory power for this study. Notably, the sample size of this study (*n* = 420) exceeds the minimum threshold of 205 samples recommended for PLS-SEM analyses. Accordingly, SmartPLS 4.0 was utilized to analyze the theoretical model, with a bootstrapping resampling procedure (5,000 resamples) employed to assess the statistical significance of path coefficients.

## 4 Data analysis and results

### 4.1 Common method variance (CMV) test

Harman’s one-factor test was employed to assess common method variance (CMV) in the questionnaire data (49). The results revealed that the total variance explained by all extracted factors was 42.11%, with the variance accounted for by the first factor falling below the 50% critical threshold (50). This suggests that CMV did not constitute a major methodological concern in the present study.

### 4.2 Measurement model

Construct reliability was evaluated primarily via Cronbach’s alpha and composite reliability (CR). As presented in Table 2, all Cronbach’s alpha and CR values exceeded the 0.7 threshold, demonstrating satisfactory internal consistency of the measurement scales (51).

**Table 2:** Reliability and validity tests of the constructs.

Construct	VIF	Items	loading	Cronbach’s α	CR	AVE
Perceived competence	2.234	I believe that GenAI can provide accurate health information.	0.880	0.850	0.909	0.769
	2.144	I believe that GenAI is capable of accurately understanding my needs regarding health information.	0.882			
	1.916	I am confident about the capabilities of GenAI.	0.869			
Perceived convenience	2.071	I can use GenAI platforms to search for health information anytime and anywhere.	0.871	0.871	0.921	0.795
	2.572	I find it convenient to search for health information using GenAI platforms.	0.904			
	2.43	I can find the health information I need using GenAI platforms both quickly and easily.	0.900			
Perceived anthropomorphism	2.121	GenAI tools are natural, and do not feel fake about them	0.878	0.875	0.923	0.8
	2.469	GenAI tools are conscious of their actions.	0.893			
	2.667	GenAI tools are more humanlike and do not feel like machines.	0.912			
User trust in GenAI	2.553	GenAI is trustworthy in terms of reliability and can provide reliable health information.	0.908	0.853	0.911	0.773
	2.599	The health information provided by GenAI is honest and trustworthy.	0.907			
	1.727	GenAI will not manipulate its responses, and making health-related decisions based on GenAI will not bring negative consequences to me.	0.820			
Subjective norms	2.693	Most people I know believe that I should try using GenAI to seek health information.	0.905	0.899	0.937	0.832
	2.985	Individuals around me with health concerns believe that it is necessary for me to attempt to use GenAI to seek health information.	0.923			
	2.723	People I trust, such as family members or close friends, believe that I should attempt to utilize GenAI to seek health information.	0.908			
Digital health literacy	1.654	I am able to comprehend health information found via the internet.	0.839	0.816	0.891	0.731
	1.873	I am able to judge whether the health information found on internet is accurate or not.	0.849			
	1.988	I am able to find information on the Internet to answer the questions on healthcare or disease treatment.	0.877			
Health information seeking intention	2.137	The probability I would consider searching health information from the GenAI is high.	0.878	0.873	0.922	0.797
	2.354	If I were to search for health information, I would consider searching from the GenAI.	0.889			
	2.668	My willingness to search for health information from the GenAI is high.	0.911			

Source(s): Authors’ own work.

Convergent validity—defined as the degree to which measurement items reflect the same latent construct—is typically evaluated via the average variance extracted (AVE) and standardized factor loadings (52). The results indicated that all item standardized factor loadings exceeded the 0.7 threshold, satisfying the fundamental psychometric criterion. Additionally, all AVE values surpassed the 0.5 benchmark, confirming sufficient convergent validity for the constructs.

Discriminant validity was primarily examined using the heterotrait-monotrait ratio (HTMT) and the Fornell-Larcker criterion. As presented in Table 3, the square root of the AVE for each construct was greater than its correlation coefficients with all other constructs (53). Furthermore, the HTMT ratios for all construct pairs fell below the 0.90 cutoff value (54, 55). Collectively, these results demonstrated that all constructs in the model exhibited satisfactory discriminant validity.

**Table 3:** Heterotrait-monotrait ratio (HTMT) and Fornell-Larcker criterion.

	1.	2.	3.	4.	5.	6.	7.
1. Digital health literacy	**0.855**	0.696	0.475	0.593	0.618	0.630	0.601
2. Health information seeking intention	0.823 [0.753, 0.887]	**0.893**	0.625	0.680	0.685	0.705	0.700
3. Perceived anthropomorphism	0.561 [0.463, 0.648]	0.714 [0.651, 0.769]	**0.894**	0.644	0.634	0.665	0.704
4. Perceived competence	0.709 [0.634, 0.777]	0.787 [0.725, 0.841]	0.745 [0.677, 0.802]	**0.877**	0.734	0.661	0.694
5. Perceived convenience	0.731 [0.649, 0.804]	0.786 [0.734, 0.835]	0.726 [0.661, 0.785]	0.851 [0.801, 0.898]	**0.892**	0.662	0.652
6. Subjective norms	0.733 [0.661, 0.797]	0.795 [0.741, 0.845]	0.749 [0.688, 0.806]	0.754 [0.697, 0.807]	0.747 [0.688, 0.801]	**0.912**	0.728
7. User trust in GenAI	0.716 [0.626, 0.797]	0.808 [0.761, 0.853]	0.816 [0.755, 0.867]	0.807 [0.746, 0.862]	0.747 [0.673, 0.815]	0.831 [0.773, 0.883]	**0.879**

The diagonal and upper diagonal show the Fornell-Larcker criterion results, while the lower diagonal shows the HTMT results. The diagonal (bold) elements are the square roots of AVEs.

Source(s): Authors’ own work.

### 4.3 Assessment of path relationships

The structural model test results revealed that trust in GenAI (*β* = 0.167, *p* = 0.001) and subjective norms (*β* = 0.162, *p* = 0.002) exerted a positive effect on HISI. Additionally, perceived competence (*β* = 0.314, *p* < 0.001), perceived convenience (*β* = 0.127, *p* = 0.001), and perceived anthropomorphism (*β* = 0.393, *p* < 0.001) positively influenced users’ trust in GenAI. Digital health literacy (*β* = 0.271, *p* < 0.001) also had a positive impact on HISI. Moreover, perceived competence (*β* = 0.249, *p* < 0.001), perceived convenience (*β* = 0.266, *p* < 0.001), and perceived anthropomorphism (*β* = 0.336, *p* < 0.001) positively predicted subjective norms. Furthermore, perceived competence (*β* = 0.120, *p* = 0.016), perceived convenience (*β* = 0.129, *p* = 0.004), and perceived anthropomorphism (*β* = 0.093, *p* = 0.021) directly affected HISI, thereby providing support for H1, H2, and H3. Moreover, we controlled for the effects of demographic variables on HISI. The results show that age (*β* = 0.060, *p* = 0.013) and usage frequency (*β* = 0.113, *p* < 0.001) have a positive impact on HISI, whereas education level (*β* = −0.013, *p* = 0.326), health status (*β* = 0.020, *p* = 0.235), and income level (*β* = 0.010, *p* = 0.337) exert no significant influence on HISI.

### 4.4 Mediating effects of trust and subjective norm

Mediating effects were examined via the bootstrapping resampling method, with the significance of indirect effects evaluated by means of 95% confidence intervals and *t*-values (56). Table 4 reports the specific indirect, direct, total direct, and total effects of perceived competence, perceived convenience, and perceived anthropomorphism on HISI. All *t*-values exceeded the critical threshold of 1.96, indicating statistical significance. Specifically, trust in GenAI exerted a significant mediating effect between perceived competence (*β* = 0.053, *p* = 0.004), perceived convenience (*β* = 0.029, *p* = 0.012), perceived anthropomorphism (*β* = 0.066, *p* = 0.003), and HISI, thereby supporting Hypotheses H4a, H4b, and H4c. Additionally, subjective norms played a significant mediating role between perceived competence (*β* = 0.040, *p* = 0.008), perceived convenience (*β* = 0.043, *p* = 0.005), perceived anthropomorphism (*β* = 0.054, *p* = 0.004), and HISI, which confirms the validity of Hypotheses H5a, H5b, and H5c.

**Table 4:** The results of the mediating effect.

Hypotheses and paths	Specific indirect effects			Total indirect effects			Direct effects			Total effects		
	*β*	*t*-value	Confidence intervals	*β*	*t*-value	Confidence intervals	*β*	*t*-value	Confidence intervals	*β*	*t*-value	Confidence intervals
PAN→UT → HISI	0.066**	2.757	[0.029, 0.106]	0.120***	4.308	[0.076, 0.169]	0.093*	2.207	[0.017, 0.167]	0.213***	4.574	[0.136, 0.290]
PAN→SN → HISI	0.054**	2.647	[0.022, 0.090]									
PCP → UT → HISI	0.053**	2.634	[0.022, 0.087]	0.093***	4.039	[0.057, 0.133]	0.120*	2.148	[0.025, 210]	0.213***	3.995	[0.122, 0.298]
PCP → SN → HISI	0.040**	2.417	[0.015, 0.069]									
PCV → UT → HISI	0.029*	2.267	[0.010, 0.051]	0.072***	3.543	[0.040, 0.106]	0.129**	2.654	[0.047, 0.208]	0.201***	4.132	[0.119, 0.281]
PCV → SN → HISI	0.043**	2.594	[0.017, 0.072]									

*p < 0.05; **p < 0.01; ***p < 0.001. PAN, perceived anthropomorphism; PCP, perceived competence; PCV, perceived convenience; UT, user trust in GenAI; SN, subjective norms; HISI, health information seeking intention.

Source(s): Authors’ own work.

### 4.5 Moderating effect of digital health literacy

Following the approach proposed by Chin et al. (57), we incorporated the calculated interaction terms into the structural model. Table 5 demonstrates that digital health literacy exerts a significant moderating effect on the direct relationships between the user perception and HISI. Specifically, DHL failed to significantly moderate the direct link between perceived competence and HISI. By contrast, it significantly strengthened the direct association between perceived convenience and HISI (*β* = 0.056, *p* = 0.009), as well as that between perceived anthropomorphism and HISI (*β* = 0.041 *p* = 0.040). These results thereby support Hypotheses H6b and H6c, whereas H6a is not supported.

**Table 5:** The results of the moderating effect.

Moderator variable	Interacting	Dependent variable	*β*	*p*
DHL	DHL*PCP	ISI	0.014	0.290
DHL	DHL*PCV	ISI	0.056**	0.009
DHL	DHL*PAN	ISI	0.041*	0.040

*p < 0.05; **p < 0.01; ***p < 0.001. PAN, perceived anthropomorphism; PCP, perceived competence; PCV, perceived convenience; DHL, digital health literacy; ISI, health information seeking intention.

Source(s): Authors’ own work.

### 4.6 Predictive relevance

Cross-validated redundancy and the coefficient of determination (*R*^2^) serve as the primary indicators of predictive relevance (52). *R*^2^ is the core index gauging the model’s overall predictive power, with *R*^2^ values >0.6, 0.3–0.6, and <0.3 indicating substantial, moderate, and weak predictive power, respectively (49). The *R*^2^ values for HISI, subjective norms, and trust were 0.685, 0.564, and 0.607, respectively, demonstrating substantial effect sizes. It is worth noting that the *R*^2^ of ISI increased by 0.014 compared with 0.671 when no control variables were included, indicating that the explanatory power of ISI was further enhanced. Stone-Geisser’s predictive relevance was evaluated via the Stone-Geisser index (*Q*^2^), which was derived using the blindfolding technique (58). *Q*^2^ value > 0 indicates acceptable predictive accuracy (52), while *Q*^2^ values > 0.35, 0.15–0.35, and 0.02–0.15 represent large, medium, and small effect sizes, respectively (59). The *Q*^2^ values for HISI, subjective norms, and trust were 0.528, 0.463, and 0.463, respectively, indicating large effect sizes. Detailed results for *R*^2^ and *Q*^2^ are presented in the following table. In addition, we examined the model fit indices. The results show that the SRMR is 0.041, which is below the threshold of 0.08. The NFI is 0.848, falling between 0.8 and 0.9. Therefore, the model exhibits an acceptable level of fit.

## 5 Discussion

Drawing on technology acceptance and behavioral intention theories, this study investigates the impacts of perceived competence, perceived convenience, and perceived anthropomorphism on users’ HISI. Key findings are elaborated as follows:

First, perceived competence, perceived convenience, and perceived anthropomorphism all exert significant positive effects on HISI. Perceived competence is defined as users’ perceptions of the accuracy and effectiveness of health information generated by GenAI. Only when users perceive GenAI as a provider of professional, accurate, and reliable health information will they form strong usage intentions and regard it as a core channel for health information acquisition. In the technology acceptance model, perceived usefulness is a pivotal determinant of technology adoption, and the positive effect of perceived competence on HISI aligns with this theoretical tenet. Perceived convenience acts as a critical driver of users’ behavioral intentions toward digital health services. In comparison with traditional medical consultations, health apps, and conventional search engines, GenAI offers instantaneity and interactivity, particularly through one-stop response functionality, which substantially reduces the time and effort costs associated with health information seeking. The stronger users’ perceived convenience, the higher their HISI—a finding consistent with the results of Ma et al. (60). Distinct from perceived competence and perceived convenience, perceived anthropomorphism represents a core attribute that differentiates GenAI from traditional artificial intelligence systems. GenAI’s anthropomorphic features, including personalized responses and natural language interaction, facilitate human-computer interaction and foster emotional connections (61). Such affective communication mimics real interpersonal interaction experiences, mitigating the sense of distance and unfamiliarity often associated with accessing health information via digital tools. This anthropomorphic interaction mode consequently enhances users’ HISI, which is in line with the findings of Kim et al. (7).

Second, perceived competence, perceived convenience, and perceived anthropomorphism exert indirect effects on HISI via trust in GenA and subjective norms. Specifically, on the one hand, users’ positive perceptions of GenAI’s competence, convenience, and anthropomorphic attributes can be effectively translated into trust in GenAI, thereby converting perceptual-level positive evaluations into actual usage intention. This finding confirms the pivotal role of trust in shaping HISI. An anonymous survey of Chinese healthcare consumers by Guo et al. (62) further supports this, demonstrating that trust in GenAI is significantly correlated with the intention to adopt GenAI for health information seeking. On the other hand, the mediating effect of SN indicates that the more positive users’ perceptions of GenAI’s attributes are, the more susceptible they are to the attitudes of their social circle toward GenAI-based health information seeking. Consequently, they are more likely to internalize the social norm that “using GenAI for health information seeking is acceptable and reasonable,” which in turn reinforces their own HISI.

Third, digital health literacy positively moderates the direct effects of perceived convenience and perceived anthropomorphism on users’ HISI. Specifically, users with higher digital health literacy are more capable of translating their positive perceptions of GenAI into HISI. By contrast, users with low digital health literacy often lack the requisite skills to operate GenAI effectively, thus failing to leverage its convenience features or fully experience its anthropomorphic interaction modes. This deficiency constrains the driving effects of perceived convenience and perceived anthropomorphism on HISI. Notably, digital health literacy does not exert a significant moderating effect on the direct relationship between perceived competence and HISI. The plausible reason is that digital health literacy cannot bridge the professional gap in clinical judgment. Health information provided by generative artificial intelligence often involves specialized medical domains, and the verification of such information largely exceeds the competency limit of ordinary users’ digital health literacy. Accordingly, regardless of the level of digital health literacy, the strength of the influence of perceived competence on health information seeking intention remains unchanged.

## 6 Conclusions, implications and limitations

### 6.1 Conclusion

First, perceived competence, convenience, and anthropomorphism significantly and positively predict users’ health information-seeking intention via GenAI. Second, trust in GenAI and subjective norms significantly mediate the relationship between these perceived characteristics and health information-seeking intention. Third, digital health literacy moderates the direct effects of perceived convenience and anthropomorphism on such intention, but shows no significant moderation for perceived capability.

### 6.2 Implications

This study provides practical implications for GenAI platform operators, healthcare providers, and policymakers. First, given the positive effects of users’ perceived convenience, perceived anthropomorphism, and perceived competence on HISI, these perceptual factors should be properly leveraged to promote users’ health information-seeking intention toward GenAI. For GenAI platform developers, they should continuously refine the core perceived attributes of GenAI to bolster the positive antecedents of users’ HISI. Specifically, platforms may engage professional medical teams in model training and content validation to consolidate users’ perceived competence, streamline health information-seeking workflows and design personalized question templates for diverse health needs to improve information acquisition efficiency and perceived convenience, and upgrade natural language interaction systems to align with users’ health communication habits, thereby fostering a more anthropomorphic experience and lowering the perceived anthropomorphism threshold for diverse user groups.

Second, enhancing user trust in GenAI is critical to reinforcing its positive driving effect on HISI. Prior research has shown that users’ trust in GenAI-generated health information is primarily shaped by digital competence, perceived utility, and social approval (63). In addition to consolidating trust foundations via the measures to optimize core perceived attributes, platforms should improve the transparency of health information generation mechanisms, tighten privacy protection protocols, and elevate the professionalism of outputted health content. These efforts can further boost users’ trust in GenAI-enabled health services, strengthen the mediating role of trust, and ultimately translate GenAI’s inherent advantages into actual user adoption intention.

Third, stakeholders should cultivate positive social evaluations of GenAI-based health information services to exert the mediating role of subjective norms. Governments should accelerate the development of a sound regulatory framework for GenAI-enabled health information services to guide the industry’s healthy and orderly development. Relevant authorities should encourage healthcare institutions to collaborate with qualified GenAI platforms in developing professional service models, thereby enhancing the professionalism and reliability of GenAI-generated health information. For medical institutions and practitioners, it is recommended to establish a pre-check mechanism for GenAI output content, such as spot-checking generated health advice against evidence-based guidelines, to enhance perceived competence among users and ensure the clinical credibility of AI-provided information. On this basis, medical institutions and practitioners should strengthen health education initiatives, guide the public to use GenAI appropriately for basic health information queries, and clarify the technology’s reference value and applicable scope.

Fourth, given the positive moderating role of digital health literacy, targeted interventions should be implemented to enhance public digital health literacy levels. Governments and public health agencies should launch specialized digital health literacy campaigns and skills training programs, provide tailored digital health education services at the community level, offer dedicated guidance for older adults individuals and those with low educational attainment, regulate the dissemination of digital health information while strengthening platform governance, and establish a regular digital health literacy evaluation mechanism. Collectively, these efforts can enhance the public’s ability to evaluate health information, improve their efficiency in accessing health content via digital tools, and amplify the positive effects of GenAI’s perceived attributes on usage intention.

Fifth, regarding the social security system, our findings indicate that GenAI has the potential to optimize the allocation of social security resources by reducing the burden of repetitive consultations on primary care physicians. GenAI can be integrated into the hierarchical medical system as a pre-consultation and triage tool. It enables residents to obtain reliable preliminary health guidance before visiting primary care doctors, thereby reducing unnecessary outpatient visits, freeing up professional resources for complex cases, and improving the overall efficiency of the medical system. Policymakers are encouraged to pilot the inclusion of certified GenAI platforms in public health service packages, establish unified quality standards and accountability frameworks, and ensure equitable access for all groups. In this way, the advantages of GenAI can be translated into tangible benefits for residents’ well-being and the long-term sustainability of the social security system.

### 6.3 Limitations

This study has the following limitations. First, it focuses exclusively on users’ intention to seek health information via GenAI, rather than empirically verifying their actual usage behavior. Future research may explore the critical factors facilitating the intention–behavior translation to enhance the practical value of study findings. Second, the study only incorporates digital health literacy as a moderating variable, while ignoring other potential boundary conditions such as health anxiety and Internet usage experience. Future studies could further examine whether these variables exert moderating effects on the proposed model. Third, this study adopts cross-sectional self-reported data. Although Harman’s single-factor test was conducted, common method bias cannot be completely ruled out. Future research should adopt procedural controls or consider statistical remedies to address this issue. Furthermore, this study is preliminary and subject to certain limitations. The robustness of the research conclusions needs to be further explored and verified in future studies. Finally, the sample was collected exclusively from China, which entails geographic and population-specific constraints. Accordingly, the findings of this study may lack generalizability to other regions or populations. Especially, the sample includes insufficient representation of older adults and people with low educational levels, due to the characteristics of online questionnaire distribution and GenAI user groups. This makes it difficult to generalize the research findings to these two groups. Future studies should recruit more diverse participants from the general population.

## Data Availability

The raw data supporting the conclusions of this article will be made available by the authors, without undue reservation.

## Footnotes

1 Central People’s Government of the People’s Republic of China: Healthy China 2030 plan. The Communist Party of China Central Committee and the State Council have officially issued the Outline of the Healthy China 2030 Plan. https://www.gov.cn/zhengce/2016-10/25/content_5124174.htm

2 Central People’s Government of the People’s Republic of China: Opinions of the General Office of the State Council on Promoting the Development of “Internet + Healthcare.” https://www.gov.cn/zhengce/content/2018-04/28/content_5286645.htm

3
https://www.wjx.cn/
